# The Association between Tau Protein Level in Cerebrospinal Fluid and Cognitive Status: A Large-Scale Analysis of GAAIN Database

**DOI:** 10.3390/brainsci11070861

**Published:** 2021-06-29

**Authors:** Kyle Eckhoff, Robert Morris, Valeria Zuluaga, Rebecca Polsky, Feng Cheng

**Affiliations:** 1 Department of Cell Biology, Microbiology and Molecular Biology, College of Art and Science, University of South Florida, Tampa, FL 33620, USA; kyleeckhoff@usf.edu; 2 Department of Pharmaceutical Science, Taneja College of Pharmacy, University of South Florida, Tampa, FL 33613, USA; rpm4@mail.usf.edu; 3 Biomedical Sciences Program, University of South Florida, Tampa, FL 33620, USA; vdzuluaga@usf.edu (V.Z.); rpolsky@usf.edu (R.P.)

**Keywords:** GAAIN, tau protein, MMSE, phosphorylated, microarray, transcriptome

## Abstract

Alzheimer’s disease (AD) and the associated neurodegenerative dementia have become of increasing concern in healthcare. The tau protein has been considered a key hallmark of progressive neurodegeneration. In this paper, a large-scale analysis of five datasets (more than 2500 people) from the Global Alzheimer’s Association Interactive Network (GAAIN) databases was performed to investigate the association between the level of tau protein, including total tau and phosphorylated tau (p-tau), in cerebrospinal fluid (CSF) and cognitive status. Statistically significant (or marginally significant) high total tau or p-tau concentrations in CSF were observed in dementia patients compared with healthy people in all datasets. There is also a statistically significant (or marginally significant) negative correlation between p-tau concentrations in CSF and Folstein Mini-Mental State Examination (MMSE) scores. In addition, transcriptomic data derived from mouse microglial cells showed multiple genes upregulated in Toll-like receptor signaling and Alzheimer’s disease pathways, including TNF, TLR2, IL-1β, and COX subunits, suggesting that the mechanism of action that relates p-tau and MMSE scores may be through overactivation of pro-inflammatory microglial activity by Aβ peptides, TNF-mediated hyperphosphorylation of tau, and the infectious spread of pathological tau across healthy neurons. Our results not only confirmed the association between tau protein level and cognitive status in a large population but also provided useful information for the understanding of the role of tau in neurodegeneration and the development of dementia.

## 1. Introduction

Alzheimer’s disease (AD) is a neurodegenerative condition diagnosed by decline in cognitive faculties and memory. It has become one of the most common forms of cognitive impairment and is characterized by neuronal destruction [1]. With the psychological and economic toll that late-stage AD pathology can create, greater pressure is placed on early diagnosis in order to initiate early treatment. Phosphorylated tau (p-tau) has been considered to be related to progressive neurodegeneration. Tau is a highly soluble microtubule-associated protein that aids in the stabilization and integrity of healthy neurons [2]. Tau is encoded by a single gene (MAPT) located on chromosome 17 that contains 16 exons, which can be alternatively spliced to form six different isoforms [3]. In particular, inclusion of exons 2 and 3 subsequently leads to the expression of tau isoforms with 0–2 N-terminal repeats, while inclusion or exclusion of exon 10 gives rise to tau isoforms with three (3R) or four (4R) microtubule-binding repeats [3]. In physiologically healthy human brains, the ratio of 3R to 4R tau isoforms is approximately 1:1. In contrast, this ratio is frequently disrupted in many different tauopathies, including Alzheimer’s disease, progressive supranuclear palsy (PSP), and frontotemporal lobar dementia [3].

Although tau proteins can undergo a variety of post-translational modifications including glycosylation, acetylation, sumoylation, and ubiquitination, which influence tau localization patterns, the phosphorylation state of tau proteins is most associated with AD pathology [4]. Tau phosphorylation status and isoform prevalence also differ across the life course. In early development, the tau isoform, 0N3R, is exclusively expressed in a more highly phosphorylated state while all six isoforms are expressed in adults in a more hypophosphorylated state [3]. Tau proteins, regardless of isoform, have 85 distinct phosphorylation sites, of which only approximately 10 sites have been isolated in healthy brain tissue utilizing mass spectroscopy [3]. In contrast, tau proteins with 45 unique phosphorylated residues have been isolated from post-mortem AD brain tissue [5]. The phosphorylation status of tau proteins is mediated by the coordinated interplay between a variety of kinases, such as glycogen synthase kinase-3 (GSK-3), cyclin-dependent kinase 5 (cdk5), and casein kinase 1 (CK1), and phosphatases including phosphatase-1 and phosphatase-2A [3].

Tau protein abnormalities have been found to be associated with AD. In AD, aberrant kinase-phosphatase coordination leads to an abundance of insoluble hyperphosphorylated tau aggregates. Hyperphosphorylated tau disassociates from microtubules and instead interacts with other tau proteins to form neurofibrillary tangles (NFTs) that disrupt synaptic activity and neuronal signaling [2]. The progressive accumulation of NFTs results in a cascade of neuronal degradation and stifled synaptic activity, resulting in continual cognitive decline.

Although the association between tau (or phosphorylated tau) protein level and AD has been identified in some studies, there is no large-scale analysis of the relationship between them. In this study, five clinical datasets (more than 2500 people) were collected from the Global Alzheimer’s Association Interaction Network (GAAIN) and the association between cognitive impairment, and phosphorylated tau (p-tau) levels (or total tau levels) in cerebrospinal fluid (CSF) was assessed. GAAIN is a large integrated database containing over 50 cross-sectional and longitudinal studies [6], of which five datasets that recorded CSF tau test values were utilized [7]. Currently, a common test for cognitive impairment and AD diagnosis is the Folstein Mini-Mental State Examination (MMSE). The MMSE was designed as a simplistic screening tool that focuses on cognitive dysfunction rather than superficial and emotional observation. This eleven-question quiz has been widely used to quantify cognitive impairment and mental status due to its ease of administration and practicality [8]. The MMSE assigns a score ranging from 0–30, which is predicated by five areas of proper cognitive functioning including recall, language, attention, orientation, and registration, with lower scores corresponding to greater cognitive dysfunction [8]. Individuals who are assigned a score ≤ 24 are typically referred to further cognitive testing to ascertain dementia or Alzheimer’s disease status [8]. Possible confounding effects derived from age and sex differences were also assessed.

In addition, transcriptomic data were analyzed and assessed using the integrative GEO2R tool to identify and compile a list of upregulated and downregulated genes in a tau mouse model (P301S) relative to a healthy control. Thus, this study not only assessed the correlations between p-tau and total tau CSF levels with regard to MMSE-measured cognitive status in a sizable population but also analyzed possible molecular mechanisms that explain, at least in part, the role of tau in dementia and AD-related neurodegeneration.

## 2. Materials and Methods

### 2.1. Datasets

Of the approximately 50 datasets in the Global Alzheimer’s Association Interactive Network (GAAIN) database, 11 datasets have CSF total tau or phosphorylated tau testing results, which included ADNI, ADNI-ARG, ALFA, ARWIBO, BIOCARD, CBAS, DESCRIPA, EDSD, EPINETTE, INDD, and PHARMACOG. ALFA and BIOCARD only included tau (total tau or phosphorylated tau) data for healthy people (MMSE > 24). On the contrary, there are tau data for only dementia patients in two datasets, ARWIBO and EDSD. In addition, there were few (≤10) dementia patients with the tau test in ADNI-ARG and PHARMACOG. Therefore, these six datasets were excluded from the analysis. Table 1 shows the preliminary list of 11 GAAIN datasets and the ones that were ultimately included in the analysis.

The datasets of the final selection consisted of three longitudinal studies and two cross-sectional studies. The ADNI is a longitudinal study that began in 2004 and focuses on the identification of biomarkers for AD prognosis and early detection for future clinical trials. Data were collected across the United States from patients who were classified as healthy or having mild cognitive impairment or Alzheimer’s disease based on MMSE evaluation. CSF total tau and p-tau levels in this study were measured in pg/mL of concentration.

The INDD is an integrated neurodegenerative disease database created by UPENN researchers in an effort to combine information on deposits in the central nervous system with clinical diagnostics and biochemical information. Biofluids, demographic information, MMSE scores, and neuropathological features were obtained.

The CBAS focuses on the study of potential biomarkers in non-demented Czech adults (age ≥ 55) who have complained about potential cognitive dysfunction. Data include results of MRIs, PET scans, CSF, and blood analysis, as well as genetic tests. The purpose of this study was to aid in the finding of risk factors, protective factors, and biomarkers for MCI diagnosis.

EPPINETTE included data of patients from the Memory Center in The University Hospital of Geneva in Switzerland. MRI, PET, and CSF analysis along with previous history and standardized cognitive test scores were all made available in this study.

The final dataset is derived from the Development of Screening Guidelines and Criteria for Predementia Alzheimer’s Disease (DESCRIPA) study. Another wide-reaching European study of data from 11 countries with follow-up surveys focusing on medication use, comorbidities, standardized cognitive and neurophysical tests, as well as MRI and CSF analysis for biomarkers.

### 2.2. Statistical Analysis of GAAIN Data Sets

The MMSE scores from these datasets were utilized to assess the degree of cognitive impairment. Individuals who are assigned a score ≤ 24 are included in dementia patient groups. People with MMSE > 24 were selected as the control group. A logistic model embedded in the GAAIN database was first performed to test whether the total tau (or p-tau) level is significantly different among the dementia and control groups. The effects of some confounding factors such as age and biological sex [7,9] were also investigated in this paper. Age and sex were also included in the logistic model to evaluated whether there was a significant difference in age and sex between dementia and control groups. *p*-values of <0.05 were considered statistically significant.

The correlation between MMSE score and tau levels was also calculated. The analysis pertaining to MMSE vs. total tau/p-tau in CSF was created through the linear regression system of GAAIN. Correlation coefficients were taken from each study with total MMSE scores used as the dependent variable, and either total tau or phosphorylated tau as the independent variable. *p*-Values < 0.05 indicated that the correlation coefficient is not equal to 0. The correlation between MMSE vs. age was also calculated via linear regression to assess the age effect.

### 2.3. Transcriptomic Data Analysis

The mechanism of the association between tau protein level and cognitive function was explored with the transcriptomic data derived from GSE129296, in which Clec7a+ microglial transcriptome profiles were compared between wild-type mice and a P301S AD model mouse exhibiting tau pathology. The P301S is a transgenic mouse model expressing a mutant human microtubule-associated protein tau that utilizes the mouse prion protein promoter (Prnp). This is a commonly used AD mouse model that exhibits comparable tauopathy-related symptoms as those observed in human AD patients. In addition, this mouse model was chosen due to the localization of tau aggregates in the hippocampal region of the brain of the mouse, one of the first sites to experience neuronal degradation. Thus, this model can be used to assess changes in transcriptomic profiles in both the early and late stages of AD pathology when compared to a wild-type mouse.

A list of statistically significant genes was generated through the use of the integrative GEO2R data tool by comparing the transcriptomes of 12-month wild-type mice with 12-month P301S mice, which were done with 4 replicates. The cutoff of statistical significance for the upregulated and downregulated genes (P301S vs. wild type) was set as the adjusted *p*-value < 0.05 and 2-fold change. Upregulated and downregulated gene lists were then inputted into DAVID Functional Annotation Bioinformatics Microarray analysis separately in order to identify enriched KEGG (Kyoto Encyclopedia of Genes and Genomes) pathways in these regulated genes [10,11,12].

## 3. Results

### 3.1. The Significant Differences of CSF Total Tau Level between Dementia Patients and Healthy People

As shown in Table 2, significant differences in CSF total tau levels between patients with dementia and healthy patients, as measured by MMSE, were observed in each of the 5 datasets. In the ADNI dataset, the average total tau readings were 272.54 pg/mL in the healthy group (*n* = 1063) and 366.39 pg/mL in the dementia group (*n* = 194; *p* < 0.001). A statistically significant difference in gender distribution was observed between the healthy group (*n* = 492 females) and the group with dementia (*n* = 73 females; *p* = 0.01). A statistically significant (*p* < 0.001) difference in age was also observed in which the average age for the healthy group was 72.94 and the average age for the dementia group was 75.31.

In the INDD dataset, the average total tau readings were 206.89 pg/mL in the healthy group (*n* = 520) and 319.30 pg/mL in the dementia group (*n* = 506; *p* < 0.001). A statistically significant difference in gender distribution was observed between the healthy group (*n* = 258 females) and the group with dementia (*n* = 298 females; *p* = 0.005). A statistically significant (*p* = 0.02) difference in age was also observed: the average age for the healthy group was 69.76 and the average age for the dementia group was 71.17.

For the CBAS dataset, the average total tau readings were 433.54 pg/mL in the healthy group (*n* = 108) and 588.76 pg/mL in the dementia group (*n* = 104; *p* = 0.02). A statistically significant difference in gender distribution was not observed between the healthy group (*n* = 58 females) and the group with dementia (*n* = 55 females; *p* = 0.78). Similarly, no statistically significant (*p* = 0.65) difference in age was observed: the average age for the healthy group was 67.01 and the average age for the dementia group was 67.75.

For the DESCRIPA dataset, the average total tau readings were 396.79 pg/mL in the healthy group (*n* = 154) and 644.13 pg/mL in the dementia group (*n* = 22; *p* < 0.001). A statistically significant difference in gender distribution was not observed between the healthy group (*n* = 70 females) and the group with dementia (*n* = 14 females; *p* = 0.46). However, a statistically significant (*p* = 0.01) difference in age was observed: the average age for the healthy group was 67.76, and the average age for the dementia group was 73.15.

For the EPINETTE dataset, the average total tau readings were 475.19 pg/mL in the healthy group (*n* = 67) and 669.30 pg/mL in the dementia group (*n* = 47; *p* = 0.02). No statistically significant difference was found for age (*p* = 0.83) and biological sex (0.41) between healthy people and patients with dementia.

Thus, there was a statistically significant association between CSF total tau and dementia status across all five datasets; however, a consistent association between possible confounders, age and sex, with regard to total tau was not observed across each of the five datasets.

### 3.2. The Significant Differences of CSF P-Tau Level between Dementia Patients and Healthy People

The difference in CSF p-tau levels between healthy patients and individuals classified as having dementia was also accessed (Table 3). For the ADNI dataset, the average p-tau readings were 26.06 pg/mL in the healthy group (*n* = 1063) and 36.41 pg/mL in the dementia group (*n* = 194; *p* < 0.001). A statistically significant difference in gender distribution was observed between the healthy group (*n* = 492 females) and the group with dementia (*n* = 73 females; *p* = 0.01). In addition, a statistically significant (*p* < 0.001) difference in age was observed in which the average age for the healthy group was 72.94, and the average age for the dementia group was 75.31.

For the INDD dataset, the average p-tau readings were 41.65 pg/mL in the healthy group (*n* = 502) and 49.49 pg/mL in the dementia group (*n* = 468; *p* = 0.002). A statistically significant difference in gender distribution was observed between the healthy group (*n* = 250 females) and the group with dementia (*n* = 273 females; *p* = 0.001). A statistically significant (*p* = 0.01) difference in age was also observed in which the average age for the healthy group was 69.91, and the average age for the dementia group was 71.18.

For the CBAS dataset, a marginal significance (*p* = 0.08) was observed for CSF p-tau level between the healthy and dementia groups. The average p-tau readings were 58.83 pg/mL in the healthy group (*n* = 103) and 68.71 pg/mL in the dementia group (*n* = 104). No statistically significant difference was found for age (*p* = 0.93) and biological sex (0.93) between healthy people and patients with dementia.

In the DESCRIPA dataset, the average total tau readings were 68.10 pg/mL in the healthy group (*n* = 154) and 99.14 pg/mL in the dementia group (*n* = 22; *p* = 0.006). A statistically significant difference in gender distribution was not observed between the healthy group (*n* = 70) and the group with dementia (*n* = 14 females; *p* = 0.21). However, a statistically significant (*p* = 0.005) difference in age was observed in which the average age for the healthy group was 67.76 and the average age for the dementia group was 73.15.

For the EPINETTE dataset, the average p-tau readings were 70.69 pg/mL in the healthy group (*n* = 65) and 90.76 pg/mL in the dementia group (*n* = 45; *p* = 0.02). No statistically significant difference was found for age (*p* = 0.84) and biological sex (0.48) between healthy patients and patients with dementia.

In summary, in these five datasets, a statistically significant association between CSF p-tau and dementia status was observed in four datasets and a marginal significance is shown in one dataset, CBAS (*p* = 0.08). There was no consistent association between possible confounders, age and sex, with regard to p-tau was not observed across each of the five datasets.

### 3.3. The Correlation between CSF Tau (Total Tau or P-Tau) Level and Cognitive Function

**Figure 1:**
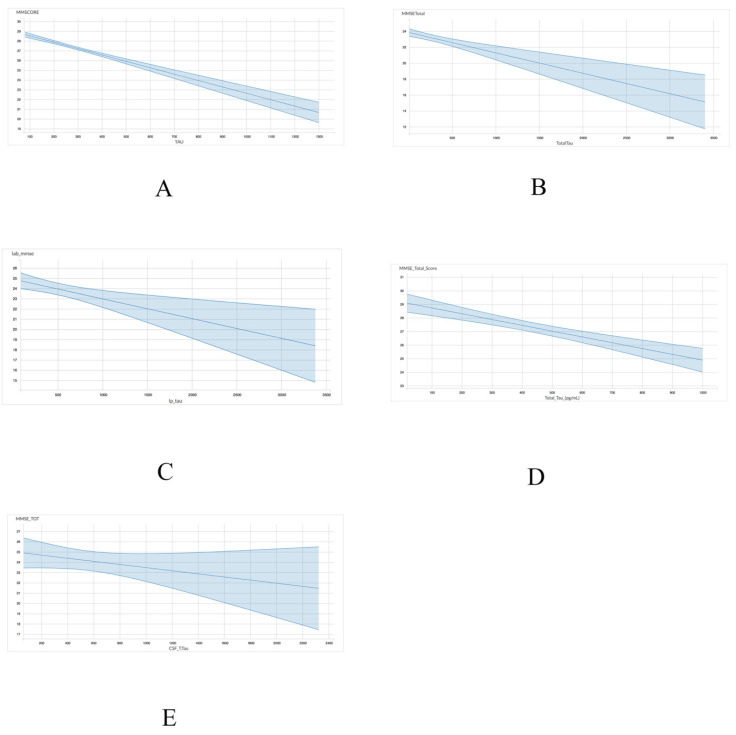
Graphical representation for the correlations between total tau and MMSE in 5 datasets from GAAIN database. (**A**) ANDI, (**B**) INDD, (**C**) CBAS, (**D**) DESCRIPA, and (**E**) EPINETTE.

A linear regression model was utilized to assess the association between either total tau or p-tau levels with MMSE scores. With regards to the association between MMSE scores and total tau, four of the five datasets, including ADNI (r = −0.006555; *p* < 0.001), INDD (r = −0.002758; *p* < 0.001), CBAS (r = −0.00193; *p* = 0.002), and DESCRIPA (r = −0.004272; *p* < 0.001) exhibited a statistically significant negative correlation (Table 4 and Figure 1). The EPINETTE dataset also exhibited a negative correlation (r = −0.001594), but did not achieve statistical significance (*p* = 0.15).

**Figure 2:**
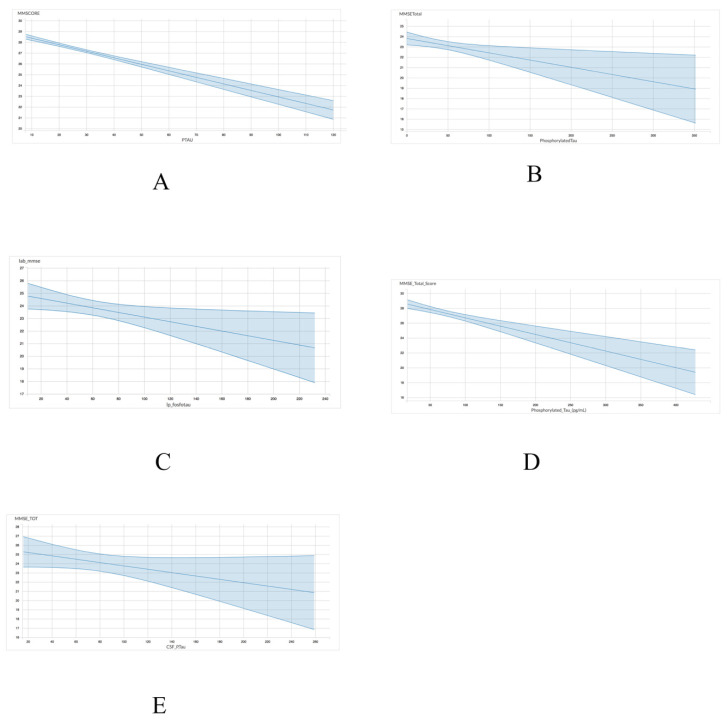
Graphical representation for the correlations between p-tau and MMSE in 5 datasets from GAAIN database. (**A**) ANDI, (**B**) INDD, (**C**) CBAS, (**D**) DESCRIPA, and (**E**) EPINETTE.

As shown in Table 4 and Figure 2, the association between p-tau and MMSE scores exhibited a comparable trend to the one observed between total tau and MMSE scores. Four of the five datasets including ADNI (r = −0.06049; *p* < 0.001), INDD (r = −0.01397; *p* = 0.01), CBAS (r = −0.0141; *p* = 0.05), and DESCRIPA (r = −0.02233; *p* < 0.001) exhibited a statistically significant negative correlation. However, the EPINETTE dataset exhibited a marginally significant negative correlation (r = −0.1821; *p* = 0.10) between MMSE scores and p-tau. Thus, p-tau may be a more consistent biomarker for identifying individuals in the early stages of dementia.

The correlation between MMSE and age was also assessed using a linear regression model (Table 4). A statistically significant negative association was found for the ADNI (r = −0.05386; *p* < 0.001) and DESCRIPA (r = −0.0944; *p* < 0.001), while the association between MMSE and age in the INDD (r = −0.01726; *p* = 0.43), CBAS (r = 0.01125; *p* = 0.72), and EPINETTE (r = 0.005968; *p* = 0.92) did not achieve statistical significance. In addition, a positive correlation between MMSE and age was observed for the CBAS and EPINETTE datasets, albeit not statistically significant.

### 3.4. Transcriptomic Data Analysis

In the 12-month P301S mouse model, 550 gene probes were shown to be significantly upregulated (adjusted *p*-value < 0.05 and 2-fold difference), and 310 gene probes were shown to be significantly downregulated relative to the 12-month wild type mice. Upregulated genes mapped to 30 distinct KEGG pathways (shown in Table S1), and downregulated differentially expressed genes did not reach statistical significance (adjusted *p* < 0.05) when mapped to any KEGG pathways. Two KEGG pathways, Alzheimer’s disease and Toll-like receptor signaling, are related to dementia. In the Alzheimer’s disease pathway, 23 genes were mapped to the Alzheimer’s disease pathway including ATP synthase, H+ transporting, mitochondrial F0 complex subunits C1 (Atp5g1), C2 (Atp5g2), C3 (Atp5g3), F (Atp5j), epsilon (Atp5e), NADH dehydrogenase 1 alpha subcomplexes 11 (Ndufa11), 1 (ndufa1), 13 (ndufa13), NADH dehydrogenase 1 beta subcomplexes 2 (Ndufb2), 5 (Ndufb5), NADH dehydrogenase 1 unknown subcomplex (Ndufc1), and NADH dehydrogenase flavoprotein subcomplex (Ndufv3). In addition, apolipoprotein e (APOE), calmodulin 3 (Calm3), cytochrome c oxidase subunits VIIa 2 (Cox7a2), VIIa 2 polypeptide 2-like (Cox7a2l), VIIb (Cox7b), VIa polypeptide 2 (Cox6a2), VI polypeptide 1 (Cox6a1), interleukin 1 beta (IL-1β), tumor necrosis factor (TNF), ubiquinol cytochrome c reductase complex III subunits X (Uqcr10) and XI (Uqcr11) were also mapped to the Alzheimer’s pathway. CD14 antigen (Cd14), chemokine ligands 3 (Ccl3), 4 (Ccl4), 5 (Ccl5), 9 (Cxcl9), 10 (Cxcl10), interferon regulatory factor 7 (Irf7), and IL-1β were mapped to the Toll-like receptor signaling pathway. In addition, secreted phosphoprotein 1 (Spp1), signal transducer and activator of transcription 1 (STAT1), thymoma viral proto-oncogene 3 (Akt3), Toll-like receptors 1 and 2 (TLR1 and TLR2), and TNF were also mapped to the Toll-like receptor signaling KEGG pathway.

## 4. Discussion

This collective study elucidated a statistically significant (or marginally significant) association between CSF tau (including total tau and p-tau) readings and cognitive function across multiple datasets containing results from distinct populations across the world. A negative correlation was also found between tau level and MMSE scores, indicating an inverse relationship between tau and subsequent scoring on an MMSE test. Higher MMSE scores were associated with lower CSF tau concentrations, while lower MMSE scores were correlated with heightened p-tau concentrations. In contrast, no consistent association between CSF tau level and confounding factors (such as age and sex) was shown. Thus, CSF p-tau or total tau may be effective biomarkers for the prediction and detection of dementia status.

Of the 30 statistically significant pathways identified by KEGG analysis, the Alzheimer’s disease and Toll-like receptor signaling pathways were the most important signaling pathways that may explain how p-tau levels in spinal fluid are associated with MMSE scores, particularly in the context of the role that microglial cells play in tau pathology. Microglial cells are brain tissue-specific macrophages that are responsible for modulating neuronal activity and clearing damaged neurons from the central nervous system (CNS) [13]. Multiple studies have demonstrated the association between high microglial activity and subsequent degree of neurodegeneration in Alzheimer’s disease. For example, neurons bearing neurofibrillary tangles have been shown to be in closer proximity to activated microglial cells than healthy neurons [14]. A tauopathy mouse model in one study showed that the earliest stages of the characteristic neurodegeneration in Alzheimer’s disease included activation of microglial cells, elevated proinflammatory cytokine levels such as IL-1β, and neuronal damage [15]. These aforementioned physiological changes were also shown to precede tau aggregation and subsequent tau pathology [15]. Thus, this manuscript proposes a possible mechanism that delineates the progression of p-tau aggregation and its subsequent deleterious effects on cognitive function as measured by MMSE.

**Figure 3:**
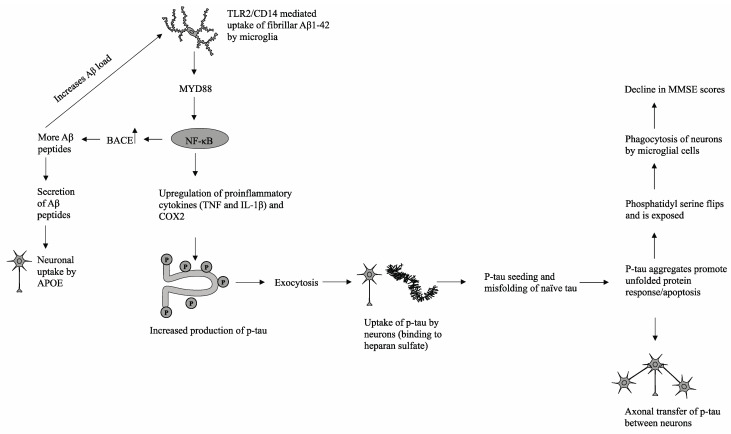
A possible mechanism that delineates the progression of p-tau aggregation and its subsequent deleterious effects on cognitive function as measured by MMSE.

In the proposed model (shown in Figure 3), there is a strong interconnection between Aβ1-42 peptides, the increased generation of phosphorylated tau, and subsequent p-tau pathology. Extracellular deposits of Aβ1-42 peptides are taken up by microglial cells via TLR2/CD14-mediated phagocytosis. Previous in vivo studies have demonstrated the interaction between TLR2 and Aβ1-42 peptides in the promotion of proinflammatory cytokine production, while ablation of TLR2-mediated signaling was shown to modulate microglial activity and reduce neuroinflammation [16]. Downstream signaling is mediated by the immune adaptor protein MyD88, which has been shown to be upregulated along with TLR2 in hippocampal tissue samples derived from AD patients and mouse models [16]. Subsequent upregulation of NF-κB and nuclear localization leads to overexpression of beta-secretase 1 (BACE), which cleaves amyloid precursor proteins (APP) into additional cytotoxic Aβ1-42 peptides [17,18]. The increase in the Aβ load triggers a positive feedback loop and generates a physiological state of chronic inflammation through continual upregulation of NF-κB and proliferation of proinflammatory cytokines [16]. In addition, cytotoxic Aβ peptides are secreted from microglial cells and can be phagocytosed by neighboring neuronal cells via apolipoprotein E (APOE), which facilitates successive Aβ peptide uptake [19]. NF-κB promotes elevated production of proinflammatory cytokines, including TNF and IL-1B, which subsequently trigger downstream stress-induced mitogen-activated protein kinase (MAPK) and glycogen synthase kinase 3 beta (GSK3β) signaling pathways in both microglia and neuronal cells [20]. The hyperphosphorylation of tau proteins, particularly in the C-terminal microtubule-binding repeats (MTBRs), reduces tau affinity for tubulin proteins and facilitates the formation of p-tau aggregates [13]. These p-tau aggregates are then partially degraded and secreted from microglial cells via exosomes to the extracellular space, at which point p-tau fragments may interact with heparan sulfate and infiltrate surrounding neuronal cells [21]. Once endocytosed into healthy neurons, the process of tau seeding occurs in which p-tau recruits and promotes the misfolding of naive monomeric tau proteins [22]. The influx of misfolded proteins triggers stress responses such as the unfolded protein response (UPR), resulting in disrupted mitochondrial membrane integrity, impaired energy production, and eventual apoptosis [23]. Through recognition of phosphatidylserine, a prominent indicator of an apoptotic cell, local microglial cells will phagocytose and degrade damaged neuronal cells [23].

In this mechanism, there are multiple manners in which p-tau is propagated and transferred across brain tissue. One such mechanism is the release of tau fragments into the extracellular space from apoptotic cells and/or subsequent degradation by activated microglia. A second mechanism, as mentioned previously, is the exocytosis of p-tau from activated microglial cells or direct secretion of tau by stressed neuronal cells. Finally, p-tau and physiological tau can be transferred across neighboring axons through synaptic connections [13]. Thus, microglial cells play a significant role in both the generation of chronic neuroinflammation, an environment conducive to cognitive decline and neurodegeneration, and the propagation of neurotoxic hyperphosphorylated tau.

Several points should be considered with regard to the different truncated forms of the tau protein and their distribution among the CSF and AD brain. Most extracellular tau in healthy patients is found in a C-truncated form, which lacks the microtubule-binding domain required for aggregation [24]. Although the exact role of physiological extracellular tau is unclear, healthy neuronal cells have been shown to physiologically secrete tau into the extracellular space [24]. However, the majority of tau associated with AD pathology is N-truncated, thus containing the microtubule-binding domain. It is thought that Aβ pathology increases both the production of and the secretion of this N-truncated form, which in turn generates a positive feedback loop to produce more Aβ peptides. However, the P301S model does not exhibit Aβ pathology, while other AD mouse models such as the APP mutant mouse model do not exhibit tau-based pathology but have cognitive issues [25]. This discrepancy may be explained by changes to secretion systems that increase secretion of the more pathogenic N-truncated tau. Even if Aβ pathology is largely absent, more N-truncated tau can be expelled into the extracellular matrix, at which point it can spread across adjacent neurons and promote the development of AD-related cognitive complications as it progresses throughout the brain tissue. It may be the interplay of these two pathological pathways that ultimately drive cognitive collapse beyond more mild cognitive issues exhibited by individuals with only one of the pathologies. Thus, p-tau levels may be a good indicator of tauopathies in general, and more specific tests, or a combination of p-tau and Aβ tests, would improve prediction of AD in particular.

The major constituents of an MMSE test include spatial orientation, language, memory, attention, and recognition. In the case of Alzheimer’s disease, the temporal lobe, particularly the hippocampus, is the origin of AD pathology [23]. The hippocampus plays a crucial role in memory and language, thus justifying the association between elevated p-tau levels and lower MMSE scores [23]. In addition, the progression of Alzheimer’s disease follows a predictable path in which it originates in the medial temporal and spreads to the lateral temporal lobe, parietal lobe, and then the frontal lobe in progressive order [26]. As the lateral and parietal lobes influence perception, spatial orientation, and language, it is reasonable to conclude that the propagation and expansion of p-tau across healthy brain tissue strongly coincides with the decline in cognitive performance and subsequent decrease in MMSE scores.

Although this GAAIN data and transcriptomic data analyses suggest that there is a strong association between CSF p-tau levels and MMSE scores, some limitations are inherent in this study. First, the sample sizes of some datasets, particularly for EPINETTE, are relatively small despite demonstrating comparable negative correlations with the other four GAAIN datasets. EPINETTE was of marginal statistical significance for p-tau and no significance for total tau in correlation analysis. The small size of the dataset could be an important reason to explain the insignificance. Increasing sample size would be ideal to further confirm the association for the dataset. Second, significant batch effects were observed across all five datasets. There was a large difference in total tau and p-tau scales across each of the studies. The ADNI dataset showed the lowest tau level (for example, total tau value is 366.39 pg/mL for patients with dementia), and DESCRIPA or EPINETTE reported the highest tau level (total tau values are more than 640 pg/mL in dementia group) in their tests. Thus, pooled GAAIN datasets are problematic for further larger-scale analysis. An additional limitation is one inherent to observation studies, of which a strong association between CSF tau levels and MMSE scores seems apparent, but a causal relationship between the two cannot be established. As the raw GAAIN data was unavailable, the embedded GAAIN tools had to be used to analyze the data. Thus, an additional limitation is the inability to generate a regression model with more than one independent variable, thus necessitating the use of individual regression models. Finally, the proposed biological mechanism requires further in vivo biological validation, despite substantial literature support, in order to strengthen the proposed physiological explanation for the observed association.

## 5. Conclusions

Given the importance of early detection of neurodegenerative diseases such as AD, there is an increased need for early markers of cognitive decline and a correlation between these determinants. Here, we presented a significant (or marginally significant) association between MMSE scores and tau (including p-tau and total tau) concentrations across five datasets. A negative correlation was also found between tau level and MMSE scores. Supplement transcriptomic data from P301S mouse microglial cells indicated 30 KEGG pathways that were differentially up-regulated compared with wild-type mice, most notably Alzheimer’s disease and Toll-like receptor signaling. Microglial uptake of cytotoxic Aβ peptides generates a positive feedback loop through upregulated NF-κB activity, giving rise to elevated Aβ peptide levels that can be secreted and taken up by surrounding neuronal cells. In addition, overstimulation of NF-κB signaling leads to the overproduction of hyperphosphorylated tau, subsequent exocytosis of tau fragments into the extracellular space, and uptake by neuronal cells. Although this study ascertains an association between CSF tau readings and cognitive status, future studies are necessary to prove a causal relationship between the two factors.

## Figures and Tables

**Table 1:** The 11 preliminary datasets derived from the GAAIN database are listed. The ALFA and BIOCARD only included tau (total tau or phosphorylated tau) data for healthy people (MMSE > 24). On the contrary, there are tau data for only dementia patients in two datasets, ARWIBO and EDSD. In addition, there were few (10) dementia patients with tau tests in ADNI-ARG and PHARMACOG. Therefore, these six datasets were excluded from the analysis.

Datasets	Full Name/Information	Data Type	Inclusion (Y/N)	Reason for Exclusion
ADNI	The Alzheimer’s Disease Neuroimaging Initiative	Longitudinal	Y	N/A
INDD	The Integrated Neurodegenerative Disease Database	Longitudinal	Y	N/A
CBAS	The Czech Brain Aging Study	Longitudinal	Y	N/A
DESCRIPA	Development of Screening Guidelines and Criteria for Predementia Alzheimer’s Disease	Cross-sectional	Y	N/A
EPINETTE	The database of patients consulting the memory center of the University hospital HUG in Geneva Switzerland	Cross-sectional	Y	N/A
ARWIBO	The Alzheimer’s Disease Repository Without Borders	Longitudinal	N	Tau value is only available for dementia patients
EDSD	European Diffusion Tensor Imaging Study in Dementia	Longitudinal	N	Tau value is only available for dementia patients
PHARMACOG	The Prediction of Cognitive Properties of New Drug Candidates for Neurodegenerative Diseases in Early Clinical Development	Longitudinal	N	There are few (≤10) dementia patients with tau test
ADNI-ARG	ADNI Argentina	Longitudinal	N	There are few (≤10) dementia patients with tau test
BIOCARD	Predictors of Cognitive Decline Among Normal Individuals	Longitudinal	N	Tau value is only available for healthy people
ALFA	TriBEKa ALFA+, a research platform to identify early pathophysiological characteristics of Alzheimer’s disease	Longitudinal	N	Tau value is only available for healthy people

**Table 2:** Difference of CSF total tau level between dementia patients and healthy people in all 5 datasets. *p*-Values < 0.05 indicate that the difference is significant.

Datasets	Parameters	Normal Group (MMSE ≥ 25)	Dementia Group (MMSE < 25)	*p*-Value
ADNI	Number of people	1063	194	
	Sex (number of female)	492	73	0.01
	Age	72.94	75.31	*p* < 0.001
	Total tau (pg/mL)	272.54	366.39	*p* < 0.001
INDD	Number of people	520	506	
	Sex (number of female)	258	298	0.005
	Age	69.76	71.17	0.02
	Total tau	206.89	319.3	*p* < 0.001
CBAS	Number of people	108	104	
	Sex (number of female)	58	55	0.78
	Age	67.01	67.75	0.65
	Total tau (pg/mL)	433.54	588.76	0.02
DESCRIPA	Number of people	154	22	
	Sex (number of female)	70	14	0.46
	Age	67.76	73.15	0.01
	Total tau (pg/mL)	396.79	644.13	*p* < 0.001
EPINETTE	Number of people	67	47	
	Sex (number of female)	41	26	0.41
	Age	71.24	70.57	0.83
	Total tau (pg/mL)	475.19	669.3	0.02

**Table 3:** Differences of CSF p-tau level between dementia patients and healthy people in all 5 datasets. *p*-Values < 0.05 indicated that the difference is significant.

Datasets	Parameters	Normal Group (MMSE ≥ 25)	Dementia Group (MMSE < 25)	*p*-Value
ADNI	Number of people	1063	194	
	Sex (number of female)	492	73	0.01
	Age	72.94	75.31	*p* < 0.001
	P-tau (pg/mL)	26.06	36.41	*p* < 0.001
INDD	Number of people	502	468	
	Sex (number of female)	250	283	0.001
	Age	69.91	71.18	0.02
	P-tau	41.65	49.49	0.002
CBAS	Number of people	103	104	
	Sex (number of female)	55	56	0.93
	Age	67.66	67.92	0.93
	P-tau (pg/mL)	58.53	68.71	0.08
DESCRIPA	Number of people	154	22	
	Sex (number of female)	70	14	0.21
	Age	67.76	73.15	0.005
	P-tau (pg/mL)	68.1	99.14	0.006
EPINETTE	Number of people	65	45	
	Sex (number of female)	40	25	0.48
	Age	70.98	70.96	0.84
	P-tau (ng/L)	70.69	90.76	0.02

**Table 4:** The correlation between MMSE and CSF total tau level, p-tau level, or age for 5 datasets from the GAAIN database. *p*-Values < 0.05 indicated that the correlation coefficient is not equal to 0.

Datasets	Number of Samples	Correlation Coefficient	*p*-Value
**Total Tau**			
ADNI	1257	−0.006555	<0.001
INDD	1026	−0.002758	<0.001
CBAS	204	−0.00193	0.002
DESCRIPA	176	−0.004272	<0.001
EPINETTE	114	−0.001594	0.15
**P-tau**			
ADNI	1257	−0.06049	<0.001
INDD	970	−0.01397	0.01
CBAS	207	−0.0185	0.02
DESCRIPA	176	−0.02233	<0.001
EPINETTE	110	−0.01821	0.1
**Age**			
ADNI	1257	−0.05386	<0.001
INDD	970	−0.01726	0.43
CBAS	207	0.01125	0.72
DESCRIPA	176	−0.0944	<0.001
EPINETTE	110	0.005968	0.92

## Data Availability

All data can be accessed through The Global Alzheimer’s Association Interactive Network (GAAIN) webpage at www.gain.org (accessed on 28 June 2021).

## Supplementary Materials

The following are available online at https://www.mdpi.com/article/10.3390/brainsci11070861/s1, Table S1: Statistically significant (FDR ≤ 0.05) pathways identified from P301S mice relative to wild-type control.

## References

[1] Mantzavinos V., Alexiou A. (2017). Biomarkers for Alzheimer’s Disease Diagnosis. Curr. Alzheimer Res..

[2] Medeiros R., Baglietto-Vargas D., LaFerla F.M. (2011). The role of tau in Alzheimer’s disease and related disorders. CNS Neurosci. Ther..

[3] Noble W., Hanger D.P., Miller C.C., Lovestone S. (2013). The importance of tau phosphorylation for neurodegenerative diseases. Front. Neurol..

[4] Trushina N.I., Bakota L., Mulkidjanian A.Y., Brandt R. (2019). The Evolution of Tau Phosphorylation and Interactions. Front. Aging Neurosci..

[5] Hanger D.P., Byers H.L., Wray S., Leung K.Y., Saxton M.J., Seereeram A., Reynolds C.H., Ward M.A., Anderton B.H. (2007). Novel phosphorylation sites in tau from Alzheimer brain support a role for casein kinase 1 in disease pathogenesis. J. Biol. Chem..

[6] Morris R., Armbruster K., Silva J., Widell D.J., Cheng F. (2020). The Association between the Usage of Non-Steroidal Anti-Inflammatory Drugs and Cognitive Status: Analysis of Longitudinal and Cross-Sectional Studies from the Global Alzheimer’s Association Interactive Network and Transcriptomic Data. Brain Sci..

[7] Toga A.W., Neu S.C., Bhatt P., Crawford K.L., Ashish N. (2016). The Global Alzheimer’s Association Interactive Network. Alzheimers Dement..

[8] Arevalo-Rodriguez I., Smailagic N., Roque I.F.M., Ciapponi A., Sanchez-Perez E., Giannakou A., Pedraza O.L., Bonfill Cosp X., Cullum S. (2015). Mini-Mental State Examination (MMSE) for the detection of Alzheimer’s disease and other dementias in people with mild cognitive impairment (MCI). Cochrane Database Syst. Rev..

[9] Tang Z., Pham M., Hao Y., Wang F., Patel D., Jean-Baptiste L., Fan L., Wang W., Wang Y., Cheng F. (2019). Sex, Age, and BMI Modulate the Association of Physical Examinations and Blood Biochemistry Parameters and NAFLD: A Retrospective Study on 1994 Cases Observed at Shuguang Hospital, China. Biomed. Res. Int..

[10] Huang D.W., Sherman B.T., Lempicki R.A. (2009). Bioinformatics enrichment tools: Paths toward the comprehensive functional analysis of large gene lists. Nucleic Acids Res..

[11] Huang D.W., Sherman B.T., Lempicki R.A. (2009). Systematic and integrative analysis of large gene lists using DAVID bioinformatics resources. Nat. Protoc..

[12] Hsu S.Y., Morris R., Cheng F. (2021). Signaling Pathways Regulated by Silica Nanoparticles. Molecules.

[13] Vogels T., Murgoci A.N., Hromadka T. (2019). Intersection of pathological tau and microglia at the synapse. Acta Neuropathol. Commun..

[14] Sheffield L.G., Marquis J.G., Berman N.E. (2000). Regional distribution of cortical microglia parallels that of neurofibrillary tangles in Alzheimer’s disease. Neurosci. Lett..

[15] Yoshiyama Y., Higuchi M., Zhang B., Huang S.M., Iwata N., Saido T.C., Maeda J., Suhara T., Trojanowski J.Q., Lee V.M. (2007). Synapse loss and microglial activation precede tangles in a P301S tauopathy mouse model. Neuron.

[16] Rangasamy S.B., Jana M., Roy A., Corbett G.T., Kundu M., Chandra S., Mondal S., Dasarathi S., Mufson E.J., Mishra R.K. (2018). Selective disruption of TLR2-MyD88 interaction inhibits inflammation and attenuates Alzheimer’s pathology. J. Clin. Investig..

[17] O’Brien R.J., Wong P.C. (2011). Amyloid precursor protein processing and Alzheimer’s disease. Annu. Rev. Neurosci..

[18] Landreth G.E., Reed-Geaghan E.G. (2009). Toll-like receptors in Alzheimer’s disease. Curr. Top. Microbiol. Immunol..

[19] Leyns C.E.G., Holtzman D.M. (2017). Glial contributions to neurodegeneration in tauopathies. Mol. Neurodegener..

[20] Zheng W.H., Bastianetto S., Mennicken F., Ma W., Kar S. (2002). Amyloid beta peptide induces tau phosphorylation and loss of cholinergic neurons in rat primary septal cultures. Neuroscience.

[21] Saman S., Kim W., Raya M., Visnick Y., Miro S., Saman S., Jackson B., McKee A.C., Alvarez V.E., Lee N.C. (2012). Exosome-associated tau is secreted in tauopathy models and is selectively phosphorylated in cerebrospinal fluid in early Alzheimer disease. J. Biol. Chem..

[22] DeVos S.L., Corjuc B.T., Oakley D.H., Nobuhara C.K., Bannon R.N., Chase A., Commins C., Gonzalez J.A., Dooley P.M., Frosch M.P. (2018). Synaptic Tau Seeding Precedes Tau Pathology in Human Alzheimer’s Disease Brain. Front. Neurosci..

[23] Kim S., Joe Y., Surh Y.J., Chung H.T. (2018). Differential Regulation of Toll-Like Receptor-Mediated Cytokine Production by Unfolded Protein Response. Oxid. Med. Cell Longev..

[24] Kanmert D., Cantlon A., Muratore C.R., Jin M., O’Malley T.T., Lee G., Young-Pearse T.L., Selkoe D.J., Walsh D.M. (2015). C-Terminally Truncated Forms of Tau, But Not Full-Length Tau or Its C-Terminal Fragments, Are Released from Neurons Independently of Cell Death. J. Neurosci..

[25] Amadoro G., Latina V., Corsetti V., Calissano P. (2020). N-terminal tau truncation in the pathogenesis of Alzheimer’s disease (AD): Developing a novel diagnostic and therapeutic approach. Biochim. Biophys. Acta Mol. Basis Dis..

[26] Raskin J., Cummings J., Hardy J., Schuh K., Dean R.A. (2015). Neurobiology of Alzheimer’s Disease: Integrated Molecular, Physiological, Anatomical, Biomarker, and Cognitive Dimensions. Curr. Alzheimer Res..

