# Supplementary material for: The Association between Tau Protein Level in Cerebrospinal Fluid and Cognitive Status: A Large-Scale Analysis of GAAIN Database
Source: Brain Sci. 2021 Jun 29;11(7):861. doi: 10.3390/brainsci11070861 (PMC8301769; doi:10.3390/brainsci11070861)
Supplement: Supplementary file 1 [file brainsci-11-00861-s001.zip › brainsci-1268097-SI.pdf]

Table S1. Statistically significant (FDR<= 0.05) pathways identified from P301S mice relative to wildtype control.

Term	Count	%	P-Value	Fold Enrichment	FDR
mmu03010:Ribosome	28	5.25	1.27E-12	5.30	2.71E-10
mmu04145:Phagosome	29	5.44	1.25E-11	4.66	1.33E-09
mmu00190:Oxidative phosphorylation	25	4.69	1.26E-10	4.94	8.97E-09
mmu05332:Graft-versus-host disease	16	3.00	2.28E-10	8.45	1.22E-08
mmu05168:Herpes simplex infection	30	5.63	2.98E-10	3.96	1.27E-08
mmu04612:Antigen processing and presentation	19	3.56	4.95E-10	6.36	1.51E-08
mmu05323:Rheumatoid arthritis	19	3.56	4.95E-10	6.36	1.51E-08
mmu04514:Cell adhesion molecules (CAMs)	25	4.69	3.22E-09	4.24	8.09E-08
mmu04940:Type I diabetes mellitus	16	3.00	3.42E-09	7.09	8.09E-08
mmu05330:Allograft rejection	15	2.81	7.29E-09	7.36	1.55E-07
mmu05010:Alzheimer's disease	23	4.32	3.69E-07	3.57	7.14E-06
mmu05320:Autoimmune thyroid disease	14	2.63	1.25E-06	5.42	2.23E-05
mmu05140:Leishmaniasis	13	2.44	2.49E-06	5.58	4.08E-05
mmu05416:Viral myocarditis	14	2.63	4.39E-06	4.87	6.68E-05
mmu05152:Tuberculosis	21	3.94	5.21E-06	3.28	7.40E-05
mmu05012:Parkinson's disease	19	3.56	6.60E-06	3.50	8.79E-05
mmu05016:Huntington's disease	22	4.13	8.99E-06	3.05	1.13E-04
mmu05310:Asthma	8	1.50	1.56E-05	9.16	1.85E-04
mmu05321:Inflammatory bowel disease (IBD)	11	2.06	4.37E-05	5.12	4.90E-04
mmu04640:Hematopoietic cell lineage	13	2.44	5.06E-05	4.20	5.38E-04
mmu05150:Staphylococcus aureus infection	10	1.88	6.48E-05	5.49	6.43E-04
mmu04620:Toll-like receptor signaling pathway	14	2.63	6.64E-05	3.81	6.43E-04
mmu05164:Influenza A	18	3.38	1.47E-04	2.89	1.36E-03
mmu04932:Non-alcoholic fatty liver disease (NAFLD)	17	3.19	1.72E-04	2.97	1.51E-03
mmu04060:Cytokine-cytokine receptor interaction	22	4.13	1.78E-04	2.50	1.51E-03
mmu04064:NF-kappa B signaling pathway	13	2.44	1.86E-04	3.68	1.52E-03
mmu05166:HTLV-I infection	23	4.32	4.15E-04	2.29	3.27E-03
mmu04672:Intestinal immune network for IgA production	8	1.50	6.95E-04	5.23	5.29E-03
mmu04142:Lysosome	13	2.44	1.51E-03	2.93	1.11E-02
mmu04062:Chemokine signaling pathway	17	3.19	1.98E-03	2.38	1.40E-02
